# Home-Based Spirometry in Patients with Interstitial Lung Diseases: A Real-Life Pilot “FACT” Study from Serbia

**DOI:** 10.3390/jpm13050793

**Published:** 2023-05-05

**Authors:** Miroslav Ilić, Jovan Javorac, Ana Milenković, Dejan Živanović, Dejan Miljković, Svetlana Kašiković Lečić, Nevena Savić, Kristina Tot Vereš, Dragica Kovačević, Emilija Vujičić, Ivan Kopitović

**Affiliations:** 1 Faculty of Medicine, University of Novi Sad, 21000 Novi Sad, Serbia; miroslav.ilic@mf.uns.ac.rs (M.I.); dejan.miljkovic@mf.uns.ac.rs (D.M.); svetlana.kasikovic-lecic@mf.uns.ac.rs (S.K.L.); ivan.kopitovic@mf.uns.ac.rs (I.K.); 2 Institute for Pulmonary Diseases of Vojvodina, 21204 Sremska Kamenica, Serbia; ana.m.jakic@gmail.com (A.M.); nenasavic@gmail.com (N.S.); krisztinatotveres@gmail.com (K.T.V.); kovacevic.dragica.ns@gmail.com (D.K.); emilija.vujicic@gmail.com (E.V.); 3 Department of Psychology, College of Social Work, 11000 Belgrade, Serbia; dejan.zivanovic@asp.edu.rs; 4 Department of Medical Sciences, College of Vocational Studies “Sirmium”, 22000 Sremska Mitrovica, Serbia

**Keywords:** interstitial lung disease, telemedicine, home-based spirometry, patients’ satisfaction, quality of life

## Abstract

(1) Background: home-based spirometry, as a form of telemedicine in pulmonology, was previously successfully implemented in clinical practice in developed countries. However, experiences from developing countries are lacking. The aim of this study was to assess the reliability and feasibility of home-based spirometry in patients with interstitial lung diseases from Serbia. (2) Methods: 10 patients were given a personal hand-held spirometer with operating instructions and asked to perform daily domiciliary spirometry for the next 24 weeks. The K-BILD questionnaire was used to assess patients’ quality of life, while the questionnaire designed specifically for this study was used to assess their attitudes toward and satisfaction with domiciliary spirometry. (3) Results: there was a significant positive correlation between office- and home-based spirometry at the beginning (r = 0.946; *p* < 0.001) and end of the study (r = 0.719; *p* = 0.019). The compliance rate was nearly 70%. The domiciliary spirometry did not affect patients’ overall quality of life or anxiety levels, as measured via different domains of the K-BILD. Patients expressed positive experiences and high satisfaction with the home spirometry program. (4) Conclusions: home-based spirometry may represent a reliable form of spirometry, exploited in routine clinical practice; however, additional research in developing countries with a larger sample size is required.

## 1. Introduction

Telemedicine is a method of providing health services that involves the use of information and communication technologies, i.e., the transmission of medically relevant information over a long distance while adhering to medical and technological standards [1]. Telemedicine is becoming increasingly popular, and its application in daily clinical practice was amplified during the COVID-19 pandemic [2,3,4], when a large number of patients suffering from chronic non-communicable diseases were denied access to health care due to their social distancing and the redistribution of health personnel to care for patients infected with COVID-19. Using telemedicine, patients were able to receive advice from their physician about the treatment of their disease, and the possibility of them being exposed to the virus during their stay in health institutions was also reduced. There are several examples of the use of information technology in respiratory medicine, such as portable pulse oximeters, physical activity trackers, and cough monitors; however, one of the most recent developments is the use of home-based spirometers in patients with chronic respiratory diseases [1] or after lung transplantation [5].

Interstitial lung diseases (ILDs) represent a diverse group of more than 200 respiratory diseases characterized through various combinations of interalveolar inflammation and fibrosis [6]. Connective tissue accumulation in the lung parenchyma causes a reduction in pulmonary compliance, which is clinically demonstrated via the onset of respiratory symptoms, particularly dry cough and dyspnea, and a restrictive pattern on pulmonary function testing. As some ILDs have a progressive course, such as idiopathic pulmonary fibrosis (IPF), serial repetition of spirometry and obtaining forced vital capacity (FVC) values are of great importance in monitoring the progression of fibrosis in these patients, the response to the applied therapy, and the prediction of unfavorable outcomes from the disease, such as acute exacerbation and mortality [7,8]. Thus, it is not surprising that the majority of ILDs clinical trials use FVC as the primary endpoint [9,10]. As the disease progresses, the burden of symptoms increases significantly, making it more difficult for some patients to attend regular medical check-ups and spirometry evaluations. Given this problem, an objective need for some type of disease monitoring at home, such as domiciliary spirometry, gained increased attention in recent years. More frequent FVC measurements, such as using home spirometry, could also reduce the size, length, and cost of clinical trials in ILD patients [11]; however, this aspect of domiciliary spirometry requires further optimization and research [12].

The first studies comparing the efficacy of home and office-based spirometers in ILDs appeared between 2016 and 2017 [7,9]. Those studies confirmed the satisfactory correlation between FVC values obtained at home and in office-based spirometry; the decline in FVC values on home spirometers proved to be a good predictor of mortality, and respondents emphasized the feasibility of this type of lung function monitoring. One of the major flaws in these studies was that the obtained spirometry data were not automatically forwarded to the health expert, but instead the patients recorded them in special diaries and brought them to their physician at the next check-up [13]. These flaws were addressed via developing a new generation of home spirometers that, using modern information technologies, can transmit spirometry results to health professionals in real-time [13,14,15,16,17].

All of the aforementioned studies utilizing home-based spirometry in ILDs patients are conducted in developed countries in Europe and North America; thus, there is a lack of studies on reliability and feasibility of home-based spirometry in underdeveloped and developing countries. Considering this problem, the aim of our study was to assess the feasibility and reliability of home-based spirometry in patients with ILDs residing in Serbia, a developing country in the western Balkans, to evaluate its effect on patients’ quality of life using the King’s Brief Interstitial Lung Disease (K-BILD) questionnaire, and investigate patients’ experiences and satisfaction with domiciliary spirometry.

## 2. Materials and Methods

### 2.1. Study Subjects

In this prospective observational pilot study, titled “Fibrosis Assessment by Clinical-functional Testing (FACT)”, 10 patients with ILDs were recruited from outpatient clinics at the Institute for Pulmonary Diseases of Vojvodina (IPDV) in Sremska Kamenica, Serbia, in 2022. Inclusion criteria were as follows: a previously established diagnosis of IPF [18] or progressive pulmonary fibrosis (PPF) in fibrotic ILDs, other than IPF, according to ATS/ERS/JRS/ALAT Clinical Practice Guideline [19]; patient age over 18; residential Internet access; and owning a smartphone or tablet. Exclusion criteria were as follows: absolute contraindications to spirometry; inability to read, speak, and/or write in Serbian; lack of residential Internet access; lack of a smartphone or tablet; and a physical or cognitive impairment that makes use of home-based spirometry or a smartphone/tablet impossible. The study was approved by the Institutional Review Board and Ethics Committee of IPDV (protocol code No. 2-II/1, date of approval 23 September 2021), while all subjects signed informed consent forms to participate in this research after being provided with information regarding the study protocol.

### 2.2. Study Design

At the baseline visit, basic socio-epidemiological characteristics and the patients’ medical history were collected. All patients underwent office-based spirometry in accordance with American Thoracic Society/European Respiratory Society (ATS/ERS) standards [20], completed the K-BILD questionnaire, and received a personal hand-held spirometer with operating instructions. The patients were asked to perform domiciliary spirometry every day for the next 24 weeks, as well as to fill out the K-BILD questionnaire once per month during the regular check-up visits. The patients underwent office-based spirometry again at the conclusion of the study, and completed a questionnaire designed specifically for this study to assess the patients’ experiences with and attitudes towards home-based spirometry, as well as their satisfaction with this type of pulmonary function testing.

### 2.3. Home-Based Spirometry

For the purposes of this study, patients received a portable ultrasonic SpiroHome Personal^®^ spirometer (Inofab Health, Ankara, Türkiye). The findings of a previous study conducted by Sekerel et al. [21] demonstrated that this spirometer met ATS/ERS performance requirements, and supported its use in clinical practice. The device communicates via Bluetooth^®^ with the appropriate SpiroHome^®^ application on the patient’s smartphone or tablet, and uses Wi-Fi for real-time data transfer to the SpiroCloud^®^ platform that could be assessed by the study’s investigators. This sort of home monitoring did not offer automatic alerts regarding the decline in lung function; however, it allowed the study team access to information on the patient’s spirometry results at any time.

At the baseline, the patients were given a 30-min training session on how to use this home spirometer and the corresponding mobile application. A healthcare professional from the study team first set up the SpiroCloud^®^ account (an online telehealth platform that collects and displays data from SpiroHome Personal^®^ spirometers in real-time). The subject was then required to download the SpiroHome^®^ application from the Android Play Store or Apple App Store. After that, they created their own SpiroHome Personal^®^ account and accepted the invitation that was sent by a SpiroCloud^®^ user to allow the healthcare professional to view the subject’s data and test results. Once the connections were made, the patients were given a verbal explanation, and watched a short video about how to use the home spirometer and the appropriate mobile application. The patient was considered educated successfully if they were able to obtain three good, reproducible FVC measurements with less than 150 mL difference between the two highest FVCs. The patients were asked to perform spirometry at home at roughly the same time every day. Spirometer data were unblinded to patients and the investigator team.

### 2.4. Patient-Reported Outcomes (PROs)

At the baseline, and every 4 weeks after that, during their visit to IPDV’s outpatient clinics, the patients were asked to complete the K-BILD health status questionnaire, which had previously been validated to assess the health-related quality of life of ILDs patients [22,23], and was used as a PROs in several studies with ILDs patients [24,25]. This questionnaire included15 questions that evaluate three aspects of the patient’s functioning: breathlessness and activities, psychological functioning, and chest symptoms. The final score on this test ranged between 0 and 100, with higher scores indicating a higher quality of life. The use of the K-BILD in our study was approved after receiving written permission from the questionnaire’s authors.

Another questionnaire was designed specifically for the purposes of this study to assess patients’ satisfaction with and attitudes toward domiciliary spirometers. After 24 weeks of research, the patients were asked to complete this questionnaire at the final visit. The questionnaire contained 20 questions, most of which (15) were positively reflected, and 5 of which (questions numbers 10, 11, 12, 14, and 15) were negatively reflected. That format is why conversion of scoring was performed for them during statistical analysis; answer number 1 on the five-point Likert scale (completely disagree) carries 5 points, while answer number 5 (completely agree) carries 1 point. The total score on this questionnaire ranged between 20 and 100, with 50 points being the median score. A score of fewer than 50 points indicated more negative attitudes among patients towards the use of home spirometers, while a score above 50 indicated more positive attitudes.

### 2.5. Statistical Analysis

Methods of descriptive and analytical statistics were used to analyze the obtained data. The data were presented as arithmetic means, standard deviations, absolute and relative frequencies, and ranges. A paired t-test was used to compare the sample means from two related groups (Initial FVC/Final FVC), while a Student’s t-test was used to compare the sample means from two independent groups (home-based FVC vs. office-based FVC). A linear trend analysis was used to assess the slope of the FVC and KBILD results from the beginning until the end of the study. The home- and office-based spirometry measurements were compared using Pearson’s correlation coefficient (r), while the same statistical analysis was used for correlations between other variables. Adherence to home spirometry was assessed through dividing the total number of home-based spirometry measurements by the total number of study days. Patient satisfaction and attitudes towards the home spirometry program were described via a home-based spirometry satisfaction survey designed for this study. Statistical analyses were performed in SPSS version 26. Statistical significance was determined at a level of *p* < 0.05.

## 3. Results

### 3.1. Patient’s Baseline Characteristics

At the time of the study initiation, 49 patients with IPF or PPF were being managed and treated at the IPDV outpatient clinic for interstitial lung diseases. Due to the availability of home spirometers, 10 out of the 16 patients who met the inclusion criteria were enrolled in the study. The ineligibility of the remaining 33 patients was primarily attributable to their lack of digital literacy, which was required for co-ordination between the home spirometer and the appropriate mobile application.

Out of ten patients with ILDs who were enrolled in this pilot study, seven patients (70%) were previously diagnosed with IPF, while the remaining three (30%) PPF patients were diagnosed with fibrotic hypersensitivity pneumonitis (accompanied by chronic obstructive pulmonary disease (COPD) in one patient). The average duration of the underlying disease was 36.6 months. Out of all patients, 6 (60%) were male, while the average age was 61.9 years. At the time of study enrollment, one patient with fibrotic hypersensitivity pneumonitis was still taking oral steroids and was shortly after switched to nintedanib, whereas other patients started the treatment with antifibrotics. Other patients’ baseline characteristics are presented in Table 1.

### 3.2. Reliability of Home-Based Spirometry

Both office- and home-based spirometry were used to measure FVC at the beginning and conclusion of the study. Even though a 3.9% decrease in mean FVC was measured using home-based spirometry between the beginning and end of the study, this difference was not statistically significant (t = 1.84; *p* = 0.09). Despite a 7.9% increase in office-based spirometry, a paired t-test revealed no significant difference in mean FVC values between the beginning and end of this trial (t = 0.83; *p* = 0.211).

Although the average FVC values of home-based spirometry were lower than the average values of office-based spirometry, there was no significant difference between them at the beginning (initial home-based FVC 74.80 ± 22.67%; initial office-based FVC 81.60 ± 24.02%; t = −0.65; *p* = 0.52) or end of the study (final home-based FVC 70.90 ± 21.44%; final office-based FVC 89.50 ± 24.02%; t = −1.59; *p* = 0.13). There was a significant positive correlation between office- and home-based spirometry at the beginning (r = 0.946; *p* < 0.001) and end of the study (r = 0.719; *p* = 0.019).

Since the results were potentially influenced by one patient with an average FVC of 29%, we decided to conduct further analysis, excluding this outlier. For the remaining nine patients, there was still no statistically significant difference between FVC at the beginning (initial home-based FVC 79.33 ± 17.52%; initial office-based FVC 86.78 ± 18.64%; t = −0.85; *p* = 0.40) and end of the study (final home-based FVC 75.89 ± 15.39%; final office-based FVC 95.56 ± 24.59%; t = −2.03; *p* = 0.06). There was still a significant positive correlation between office- and home-based spirometry at the beginning of the study (r = 0.907; *p* < 0.001), while the correlation at the end of the study was positive, albeit weak (r = 0.478; *p* = 0.19).

### 3.3. The Trend of the Average Weekly FVC of the Whole Cohort over the Examined Period and Patients’ Compliance to Domiciliary Spirometry

**Figure 1:**
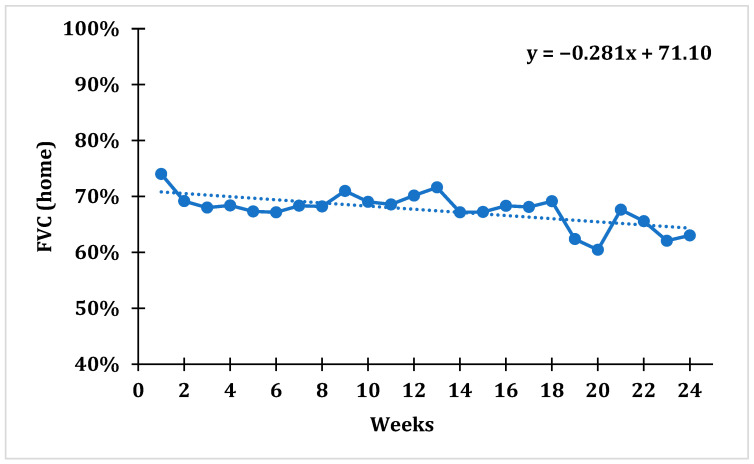
Trend for average weekly percentage of FVC (home-based spirometry) taken from all patients within 24 weeks of tracking.

After tracking average weekly FVC values using domiciliary spirometry, a statistically significant trend with a downward slope was established for the whole cohort (F = 6.833; *p* = 0.009), which indicates that the average percentage of FVC gradually decreased over time. The average weekly FVC value over the 1st week was 74.01 ± 18.85%, while over the 24th week it was 63.03 ± 23.57%, which represents a drop in FVC value of 14.83% during the course of the whole study (Figure 1).

For every patient, we calculated the average FVC value on home-based spirometry, percentage of FVC change during 24 weeks of tracking, and compliance with home-based spirometry. Overall compliance was 68.95%, ranging from 39.88% to 97.62% (Table 2).

### 3.4. Patients’ Reported Outcomes Using K-BILD

**Figure 2:**
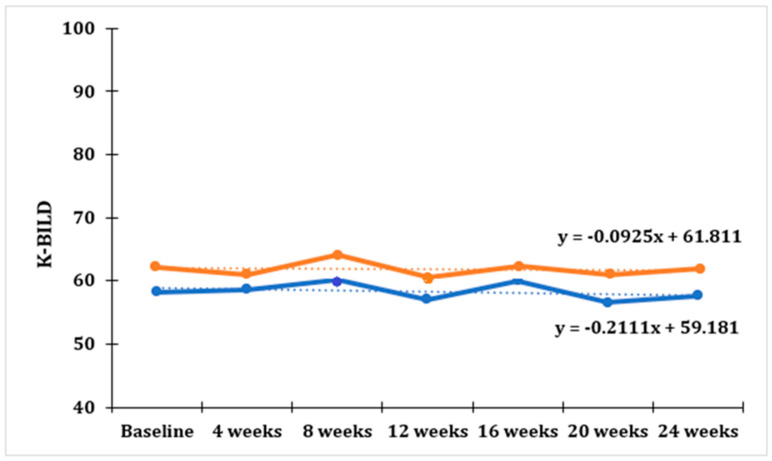
Trend for average scores of K-BILD (o) (in blue) and KBILD (pf) (in orange) taken from all patients within 24 weeks of tracking.

The average values of FVC on home-based spirometry and the average total score on the K-BILD questionnaire, as well as scores on different domains of this questionnaire (breathlessness and activities, psychological functioning, and respiratory symptoms) for the entire cohort group were measured every 4 weeks from the start of our study (baseline). No statistically significant trend was established for either the average total scores of the K-BILD questionnaire (F = 0.340; *p* = 0.574, Figure 2), or for the average scores of the psychological functioning domain within the K-BILD questionnaire (F = 0.029; *p* = 0.868, Figure 2).

### 3.5. Patient Satisfaction with Home-Based Spirometry

A home-based spirometry satisfaction survey was given to all 10 patients at the conclusion of this study. The mean satisfaction survey value for this cohort was 84.00 ± 5.44 points, with a minimum of 73 and a maximum of 94. One patient who attended elementary school had 94 points in the satisfaction survey; patients who attended high school had an average of 84.50 ± 2.59 points, while patients who attended college/university had an average of 79.67 ± 6.11 points.

This survey consisted of 20 items. As one item (Item 5) had all the same answers, this item was automatically removed from the analysis. One more item (Item 13) did not show adequate statistical saturation, and was also extracted from the final version of the questionnaire. After that, statistical testing for the internal consistency of this survey determined a Cronbach α value of 0.72, thus creating a questionnaire on user satisfaction with the home-based spirometry program consisting of 18 items. Due to the fact that this was a pilot study in a developing country, and used a small sample size, the objective statistical validation of the questionnaire should, in future studies, be conducted with a larger patient sample.

A qualitative analysis of the received responses revealed mostly positive experiences and high patient satisfaction with the home spirometry program. The great majority of patients concurred that the home spirometer and accompanying mobile application were easy to use, performing spirometry every day did not take too much time, such a program gave them an extra sense of security and medical supervision, and made it easier for them to communicate with the physician. Spirometry was not burdensome for the majority of patients. During the study period, a small number of patients experienced technical issues, mainly related to Internet and Wi-Fi connection. All patients agreed or strongly agreed that they would recommend this program to other patients and would continue to use it after the study was over.

## 4. Discussion

In this real-life pilot study, 10 patients were enrolled in the home-based spirometry program. The positive and statistically significant correlation between the FVC values obtained from home- and office-based spirometry at the beginning and end of the study suggests that home-based spirometry may be a reliable tool for pulmonary function monitoring. Using home spirometers, a 24-week follow-up revealed a trend of decreasing FVC values. Approximately 70% of patients complied with daily home-based spirometry. The home spirometry program had no effect on the quality of life or anxiety levels of patients, as measured via the various domains of the K-BILD questionnaire. Patients expressed both positive experiences and high satisfaction with the domiciliary spirometry.

Shortly after the introduction of home spirometers in clinical practice and scientific research, some of the benefits of this type of lung function monitoring were identified, such as the potential to reduce disparities in access to specialists and the economic burden of health care, improve patients’ quality of life, and avoid adverse outcomes through early detection of acute exacerbations of underlying disease [1,26]. However, one of the basic questions that needed to be addressed at the start of the use of home spirometers was the reliability of the results obtained, in comparison to those obtained during office-based spirometry. In a large number of studies conducted thus far, it was determined that there are no statistically significant differences in the values of parameters measured during spirometry in office and home conditions [5,13,14,15,16,27]. According to the findings of our study, there were no statistically significant differences between FVC values at the beginning and end of the study for both office- and hospital-based spirometry, with a statistically significant positive correlation between them. However, they must be interpreted with caution due to the small sample size.

During the 24-week follow-up, there was a tendency toward lower FVC values for the whole cohort of patients. When evaluating the percentages of FVC change during the 24 weeks of our study (Table 2), it can be seen that in three patients, an increase in FVC values was recorded, while in five patients, a decline in lung function was observed (a fall in FVC ≥ 5% more than predicted is considered significant according to the newest guidelines) [28], with the trimmed mean of FVC decline over 24 weeks being 8.3%. However, discussing such data is problematic because the study included patients with varying FVC values at the onset because they were in different stages of their disease. Even though a decline in FVC values over time is expected in patients with chronic ILDs, we did not consider those five patients as having a progressive disease due to the short duration of follow-up (approximately six months), absence of clinical and radiological progression, and consistency of scores on several domains of the K-BILD questionnaire. The possibility of sending an automated e-mail notification to the patient’s physician in the event of a significant decline in lung function would help in the earlier detection of possible adverse events, such as an exacerbation of IPF or a new infection. In some studies, including ours, lower, but statistically insignificant, values of pulmonary function parameters were obtained using home spirometry compared to clinical spirometry [13,14,29]. The possible reasons for this discrepancy may be of a technical nature, or related to decreased patient motivation to frequently perform spirometry at home, a lack of medical staff supervision, etc. We also observed that the average FVC values on the office-based spirometry increased at the end of the study. Even though the examined patients had progressive ILDs, they all started with appropriate treatment at the beginning of the study that may have influenced the results; however, the fact that office-based spirometry is performed under controlled conditions in hospitals cannot be omitted. One of the possible explanations for the FVC improvement in office-based spirometry is that patients did not achieve realistic results at the beginning of the study, and that, through performing spirometry daily at home, they enhanced their technique and developed a routine, resulting in improved FVC values at the conclusion of the investigation.

Patients were instructed to perform daily home spirometry at approximately the same time as at the start of the research. At the conclusion of the trial, we found that nearly 70% of subjects were compliant with the daily domiciliary spirometry. However, in other studies with a similar methodology, higher percentages of compliance with home spirometry were obtained, mostly above 90% [13,14,15,16]. In one pioneering study from 2017, adherence to home spirometry was observed to decrease over time [9], and the same was seen in several studies following lung transplant patients [5] or patients with Duchenne Muscular Dystrophy [30]. One of the potential reasons for this result, as well as for the slightly lower-than-expected compliance rate in our study, is that some patients forgot to perform the spirometry every day, with the majority of them being elderly and unfamiliar with the daily use of modern technology. In addition, we learned from the patients during the exit interviews that it was unsafe for them to bring spirometers on vacations or weekend trips outside the home, primarily due to the risk of device damage during transport, but also due to the possibility of a lack of Internet access. Some cultural and educational differences between patients from developed and developing countries should be considered as well when discussing the lower adherence to home-based spirometry. Some of the potential barriers for irregular measurements, in a study by Wasilevska et al. [30], were forgetting about the procedures, and having limited time to perform tests. Considering that the progression of pulmonary fibrosis is slow in the majority of ILDs, we believe that it is not necessary to perform spirometry on a daily basis in these patients. Instead, spirometry can be performed less frequently, such as once or twice per week. In the event of worsening respiratory symptoms, the patient should perform home-based spirometry more frequently, as a decline in lung function may indicate an acute exacerbation of underlying ILDs, a respiratory infection, or the exacerbation of other chronic respiratory or cardiovascular conditions (COPD, asthma, heart failure, etc.). Given that the patient’s physician has access to the results of home spirometry at all times, in the event of observed clinical deterioration, a series of therapeutic measures can be promptly implemented, even in the patient’s home settings, thus fulfilling one of telemedicine’s primary purposes: improving the quality of health care. The proposed application of home-based spirometry (twice per week with an integrated alarm within the mobile application reminding them to perform spirometry and additional tests in case of respiratory symptoms) may increase patient motivation to utilize a home spirometer. In a study of lung transplant recipients, adherence to daily pulmonary function testing was 80%, while adherence to weekly measurement was 100% [5].

The K-BILD questionnaire was used to evaluate the patients’ quality of life during the home spirometry program. Patients completed this questionnaire at the start of the study and then every 4 weeks after that. Given the small number of participants in this pilot study, it is difficult to draw definitive conclusions about the significance of the scores obtained on the K-BILD and its subscales in relation to home-based spirometry; only future studies with a larger number of participants will be able to provide definitive answers. Nevertheless, the overall scores on this questionnaire did not change significantly during the course of the patients’ follow-up period. This result could imply that the daily home spirometry had no impact on the decline in their quality of life; however, in interpreting such a result, statistical limitations due to the small sample size must be taken into account. Similar results were obtained in other studies that used the K-BILD questionnaire as a measure of the quality of life [13], as well as using other questionnaires, such as the King’s Sarcoidosis Questionnaire (KSQ) and the Euroqol-5D-5L (EQ5D-5L) [14]. In one study, however, a decrease in the overall score on the K-BILD questionnaire was observed, associated with deterioration in quality of life [16]. The K-BILD questionnaire measures a patient’s overall quality of life; however, one of its subscales—the psychological scale—is devoted to evaluating a patient’s psychological wellbeing. In a 2020 study, when scores on this K-BILD subscale were compared between a group of patients who used a home spirometer and a group of patients who received standard care, the difference in scores exceeded the minimal clinically important difference (MCID), indicating that the home spirometry program had a positive impact on the psychological wellbeing of patients [15]. In our study, no statistically significant trend was observed for the average scores of the psychological functioning domain within the K-BILD questionnaire, which could indicate stable patient anxiety levels during the home spirometry program. However, this suggestion cannot be asserted with certainty based on the small sample size.

Even though the majority of patients with ILDs treated at our Institute, or their family members, had Internet access through their home and/or smartphone, only 16 had necessary informatics literacy to be included in the study (10 were included because there were a limited number of devices available). The majority of patients that did not meet the requirements to enter the study were elderly and could manipulate only basic functions on their smartphones, while coordination between handheld spirometer and appropriate mobile application was difficult for them. All of these factors suggest that the feasibility of home-based spirometry among our patients may not be the same as in developed countries; however, with the increasing availability of modern technology, we believe that this situation will change in the coming years in developing countries. On the other hand, all patients that participated in our pilot study expressed positive experiences and high satisfaction regarding the home spirometry program. Positive experiences were recorded in the majority of other studies that addressed this issue, where different questionnaires were used to analyze patients’ satisfaction [5,13,14,15,16,27]. Although our patients had no significant problems during the study and did not find home spirometry burdensome, other studies have identified potential barriers to home-based spirometry [13,28], such as a lack of internet access, poor quality control of the spirometry, difficult manipulation ofthe handheld spirometer, a lack of motivation, home spirometry being burdensome for some patients, the occurrence of side effects after spirometry (cough, dyspnea), anxiety after seeing slightly worse results, increased frequency of depression and anxiety, poor compliance, etc.

The primary limitation of this pilot study was the small number of patients, sourced from a single tertiary care facility, who were included. Nonetheless, unlike studies with obstructive pulmonary diseases, studies with interstitial lung diseases often include a smaller number of patients because the prevalence of these disorders in the population is generally low. Another limitation is the relatively short follow-up period, which makes generalizing the obtained results difficult and reduces the possibility of identifying potential adverse events during treatment, such as an exacerbation of IPF. Furthermore, it was not possible to include all consecutive patients because most of them were elderly and did not have access to the modern technologies required for this study. Finally, we were not able to address some important clinical aspects of the diseases, such as differences in dyspnea and other symptoms between patients.

In conclusion, promising findings on the feasibility and reliability of home-based spirometry were obtained, while users of the home spirometry program reported favorable experiences and high satisfaction. Domiciliary spirometry did not affect patients’ health-related quality of life, as measured using the K-BILD survey. Since the compliance with daily home-based spirometry in ILDs patients was satisfactory, albeit lower than we expected, we propose the application of home-based spirometry twice per week with additional tests in case of respiratory symptoms. An integrated short questionnaire assessing patients’ health-related quality of life within the appropriate mobile application may also be beneficial. The results of our study suggest that home-based spirometry may be put to use in routine clinical practice as a useful form of telemedicine in pulmonology. We encourage clinicians in underdeveloped and developing countries to consider incorporating home-based spirometry into their patient management and scientific research programs.

## Figures and Tables

**Table 1:** Baseline characteristics of patients.

Variable		Patients (*n* =10)
Gender	Female Male	4 (40%) 6 (60%)
Underlying disease	IPF Fibrotic HP	7 (70%) 3 (30%)
Treatment	Pirfenidone Nintedanib	7 (70%) 3 (30%)
Smoking status	Active smoker Former smoker Non-smoker	2 (20%) 6 (20%) 2 (20%)
Formal educational level	Primary school Secondary school College Faculty	1 (10%) 6 (60%) 1 (10%) 2 (20%)
Age	Average (years) ± SD (range)	61.9 ± 7.71 (44–72)
Duration of the disease	Average (months) ± SD (range)	36.60 ± 34.69 (6–120)
Home-based spirometry (% of predicted value)	Average ± SD (range)	74.80 ± 22.67 (34–121)
Office-based spirometry (% of predicted value)	Average ± SD (range)	81.60 ± 24.02 (35–133)
K-BILD overall score	Average ± SD (range)	58.24 ± 7.87 (48.5–68.7)

IPF—idiopathic pulmonary fibrosis; HP—hypersensitivity pneumonitis; SD—standard deviation.

**Table 2:** Average FVC on home-based spirometry for each patient, percentage of FVC change during 24 weeks of tracking, and compliance with home-based spirometry.

Patient Number	FVC (Home Spirometry, %) $\bar{x}$ ± SD	Percentage of FVC Change during 24 Weeks (%)	Compliance (%)
1	74.57 ± 7.99	+10.70	77.97
2	76.69 ± 10.12	−7.69	39.88
3	114.27 ± 12.87	−0.70	41.67
4	29.18 ± 2.36	−28.78	97.62
5	57.54 ± 1.64	−4.15	81.55
6	78.21 ± 4.24	+2.69	53.57
7	82.51 ± 2.98	−8.68	94.64
8	78.24 ± 4.19	−8.55	39.88
9	72.47 ± 2.49	−5.59	57.14
10	58.83 ± 4.01	+2.17	75.00

$\bar{x}$—mean, SD—standard deviation

## Data Availability

Data sharing is not applicable to this article.
